# Is It Still Possible to Think about HSP70 as a Therapeutic Target in Onco-Hematological Diseases?

**DOI:** 10.3390/biom13040604

**Published:** 2023-03-28

**Authors:** Nayla Mouawad, Guido Capasso, Edoardo Ruggeri, Leonardo Martinello, Filippo Severin, Andrea Visentin, Monica Facco, Livio Trentin, Federica Frezzato

**Affiliations:** Hematology and Clinical Immunology Unit, Department of Medicine, University of Padova, 35128 Padua, Italy

**Keywords:** Heat Shock Proteins, HSP70, leukemia, lymphoma, myeloma, therapeutics

## Abstract

The search for molecules to be targeted that are involved in apoptosis resistance/increased survival and pathogenesis of onco-hematological malignancies is ongoing since these diseases are still not completely understood. Over the years, a good candidate has been identified in the Heat Shock Protein of 70kDa (HSP70), a molecule defined as “the most cytoprotective protein ever been described”. HSP70 is induced in response to a wide variety of physiological and environmental insults, allowing cells to survive lethal conditions. This molecular chaperone has been detected and studied in almost all the onco-hematological diseases and is also correlated to poor prognosis and resistance to therapy. In this review, we give an overview of the discoveries that have led us to consider HSP70 as a therapeutic target for mono- or combination-therapies in acute and chronic leukemias, multiple myeloma and different types of lymphomas. In this *excursus*, we will also consider HSP70 partners, such as its transcription factor HSF1 or its co-chaperones whose druggability could indirectly affect HSP70. Finally, we will try to answer the question asked in the title of this review considering that, despite the effort made by research in this field, HSP70 inhibitors never reached the clinic.

## 1. State of the Art of Treatment in Onco-Hematological Diseases

Drug resistance and increased cancer cell survival are the main burden leading to failure in anti-cancer treatment or relapse. These limitations are influenced by either innate or acquired factors. Recently, in the drug discovery field, different approaches are emerging for the identification of novel targets in onco-hematological diseases with the detection of good candidates, highlighted thanks to the interplay between medicinal chemistry and chemical biology. A few examples are given below. In the treatment of chronic myeloid leukemia (CML) and acute lymphoblastic leukemia (ALL), the development of tyrosine kinase inhibitors (TKIs) against the BCR-ABL oncogene is an effective approach. Pandrala et al., designed a novel inhibitor for CML with enhanced cardiac safety and low toxicity [1]. In acute myeloid leukemia (AML), efforts led to the discovery of a class of compounds with anti-mitotic activity based on the inhibition of tubulin polymerization [2]. These anti-tubulin agents showed promising outcomes in the treatment of refractory lymphomas [3]. However, different approaches in AML, relied on the chemical modification enhancing the proteolysis-targeting chimera and reinforcing the antiproliferative effect of the approved targeting of FLT3-ITD in clinics, designed against the most common mutations [4]. A different type of modification relied on improving the molecular dynamics to stabilize the interaction between the inhibitor and FLT3 inactive state, driving anti-proliferative activity in vitro and in vivo [5]. In diffuse large B-cell lymphoma (DLBCL), targeting BCL6, a transcriptional repressor involved in the formation of germinal centers might be a powerful tool to treat these patients [6]. In addition, small molecules were designed to target protein abundance using small mRNA splice modulation and protein degradation rather than using the inhibitory mechanism against a specific protein [7]. Other small molecules interacting with the mTOR pathway, such as icaritine analogues, bypassed the low activity and off-target limits, providing a new tool in anti-multiple myeloma (MM) therapy [8]. Chronic lymphocytic leukemia, lastly, is a good example of how translational research has brought targeted drugs to the clinic such as ibrutinib, acalabrutinib and zanubrutinib (Btk inhibitor), and venetoclax (Bcl-2 inhibitor). Thanks to the success of these drugs, novel combinations are making their way into the therapeutic scenario of CLL. To name a few of the recent discoveries in targeting CLL cells, SRX3305, a novel small-molecule that targets BTK/PI3K/BRD4 in CLL cells, disrupts CLL cell proliferation and promotes apoptosis [9]. The multi-kinase inhibitor TG02 inhibits cyclin-dependent kinase 9 blocking the activation of RNA polymerase II, thus leading to Mcl-1 depletion and, to rapid apoptosis [10]. These and other discoveries boosted the introduction of new therapeutic strategies; nevertheless, the search for new molecules to be targeted is more active than ever, especially for those patients who proved to be resistant to the available therapies.

## 2. The Heat Shock Protein of 70kDa (HSP70)

### 2.1. The Heat Shock Proteins

**Figure 1:**
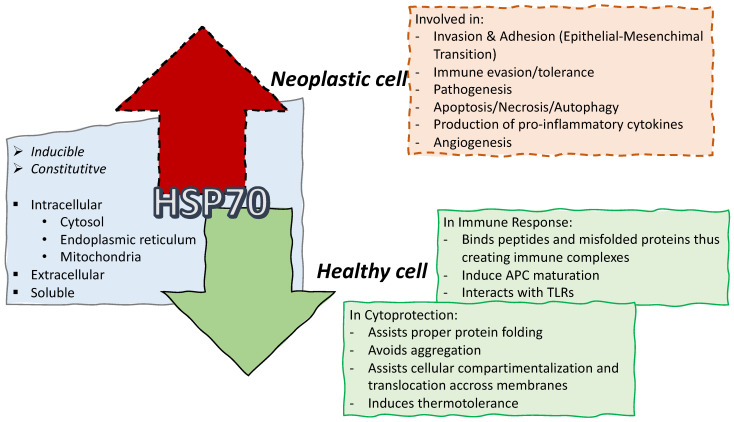
HSP70 involvement in healthy versus neoplastic cell. Different forms and localization of HSP70 are reported in the grey object. Roles of HSP70 are described in physiologically healthy cells, (green continuous line) and tumor conditions (cancerous cell, red dotted line) [11,14,16,17,18]. APC = antigen presenting cell; TLRs = toll like receptors.

Heat shock proteins (HSPs), found in all organisms and in different subcellular compartments, are a family of evolutionarily conserved and ubiquitously expressed molecular chaperones [11]. Although HSPs can be activated by different stresses that damage proteins, these proteins are so-called “heat-shock proteins” because, in 1974, the method used to upregulate their expression was the heat shock [12]. When cells undergo physiological and environmental stresses (hyperthermia, ischemia, anoxia, toxins or UV light, viral particles, surgical/emotional/mechanical stress), HSPs are upregulated [11]. HSPs are involved in physiological cellular processes, peculiarly in folding newly synthesized proteins, assembling complexes of several proteins, transporting/sorting of proteins to the correct subcellular compartments, in controlling the cell-cycle and in cell signaling. More recently, HSPs have been involved in antigen presentation to specialized cells of the immune system [13]. HSPs accumulate in high levels in numerous human cancers with roles in escaping programmed cell death, angiogenesis, invasion and metastasis [14]. Based on their relative molecular weight, mammalian HSPs are divided into families, of which the HSP70 family contains 13 members, products of the *HSPA* gene. HSP70 functions in healthy and cancerous cells are summarized in Figure 1. HSPs can also be subdivided into ATP-independent and ATP-dependent proteins based on their mechanism of action [15].

### 2.2. The HSP70 Family

The HSP70 family has been observed to be one of the most conserved protein family during evolution: it is found in all organisms, from archaebacteria and plants, to humans [12]. Eukaryotic cells have more genes encoding HSP70 and the human HSP70 family encloses at least 13 gene products which are different from each other by structure, expression and localization (Table 1). The inducible and the constitutive forms of the HSP70s, called HSP72 (or HSP70) and HSC70, respectively, are mainly expressed in the cytosol, while other members are localized in the mitochondria (mt; GRP75) or in the endoplasmic reticulum (GRP78). This family of proteins includes surface and extracellular members, it has been well established that HSP70s can also be present outside the cell acting as a signal for other cells [19,20]. Different HSP70s are also selectively expressed on the cell surface of infected or tumoral cells. Extracellular HSP70s exist: (1) embedded in the plasma membrane [21,22]; (2) associated with exosomes; (3) secreted as free soluble protein, previously thought to exclusively be released as a consequence of cell death [23,24]. As far as membrane-HSP70 is concerned, the unique sequence exposed to the extracellular milieu is the “TKD” sequence (i.e., TKDNNLLGRFELSG), that comprises aa 450-461 in the C-terminus of inducible HSP70 and is detectable with a unique monoclonal antibody named cmHSP70.1 [25]. HSP70 found on the cell surface [26,27] is present at the lipid rafts level [28]. In the extracellular medium, HSP70-containing vesicles derived from the plasma membrane have been detected, with HSP70 being exposed on the surface of these vesicles [22,29]. HSP70s have also been detected within extracellular vesicles. They are exported through the non-classical secretory pathway, mostly via exosomes [30]. As regards soluble HSP70s, they can be secreted by lysosomes, via the ABC-transporter or by secretory-like granules. HSP70s are supported by several co-chaperones (e.g., BAG family) which constitute up to 3% of the total protein amount of non-stressed cells. The co-chaperones are essential for HSP70 activity, as they initiate the binding to the client proteins [31].

### 2.3. HSP70 Structure and Chaperone Cycle

**Figure 2:**
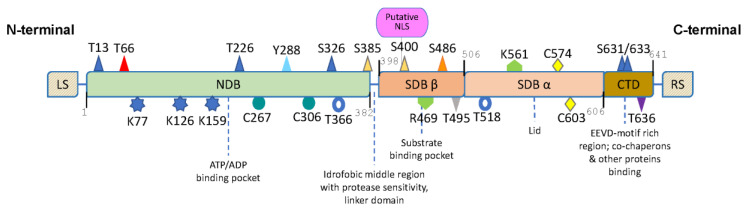
HSP70s’ structures. Principal domains and post-translational modification sites are represented. NBD = nucleotide-binding domain; SBDβ = substrate-binding domain; SBDα = helical lid domain; CTD = C-terminal domain. Numbers represent aa position. T = Threonine; K = Lysine; C = Cysteine; Y = Tyrosine; S = Serine; R = Arginine. NLS = Nuclear Localization Signal. LS domain in HSPA5 is the presumed N-terminal endoplasmic reticulum (ER) localization signal; in HSPA9 it indicates the N-terminal mitochondrial import signal. The RS domain is the ER retrieval sequence present in HSPA5. Blue triangles are phosphorylation sites for Polo-like kinase 1 (Plk) with S631 being phosphorylated also by Akt, red triangle is Nek6 phosphorylation site, light blue triangle phosphorylation is required for the binding of HSC70 to methotrexate, yellow triangles are phosphorylation sites for MAP kinases, phosphorylation of purple triangle leads to an increase in HSP70-HOP and to a decrease in HSP70–CHOP binding, the bacterial eukaryotic-like LegK4 phosphorylates grey triangle, and protein kinase A (PKA) mediates phosphorylation of orange triangle. Blue rings are AMPylation sites. Blue stars undergo acetylation. Light green arrows are sites of methylation. Green circles could be oxidized. Yellow rhombuses are glutathionylation sites [41,42,49].

HSP70 protein structure (Figure 2) consists of: (i) an N-terminal ATPase domain where ATP exchange represents the driving force inducing the conformational change required for target protein binding and release; (ii) a 15kDa substrate-binding domain (SBDβ); (iii) a further 10kDa SBDα functioning as a ‘lid’ which controls the availability of the substrate binding domain to target proteins; (iv) a C-terminal domain containing an EEVD motif for co-chaperone binding [41]. HSP70 structure contains several post-translational modification sites, such as phosphorylation, AMPylation, ADP-ribosylation, acetylation, ubiquitination, methylation, and thiol oxidation [42]. HSP70 chaperone activity undergoes a functional cycle, including the J-domain protein HSP40 and nucleotide exchange factor (NEF) co-chaperones. In the first phase (I), inactive HSP70 binds to ATP and subsequently binds transiently to the client protein stimulating ATP hydrolysis. A J protein facilitates the ATP hydrolysis, binds the client protein and presents it to HSP70. The second step (II) promotes the hydrolysis of ATP causing the undocking between the SBD and NBD of ADP-HSP70, the chaperone exhibiting high affinity for its substrate. Then (III), ADP nucleotide detaches from HSP70 while NEF remains associated with HSP70 and is thought to prevent ADP from rebinding. Once the peptide sequence has been folded (IV), a NEF (i.e., BAG1 or HSPBP1) stimulates ADP release and promotes the binding of a new ATP molecule to HSP70 within the NBD cleft, this binding causing the α-helical lid to lift, enabling the client-protein to be released and the cycle to start again [43,44,45]. In this context, we have to mention HSP110, firstly described as a co-chaperone of HSP70 and HSC70 and then characterized as the most abundant NEFs of HSP70, known to recognize and bind denatured or misfolded proteins for other HSPs. Together with HSP70 and HSP40, the HSP110–HSP70–HSP40 complex is an important chaperone network that disaggregates and refolds aggregates of denatured proteins [46,47,48].

## 3. HSP70 and Its Targeting in Onco-Hematological Diseases

While in normal cells HSP70 has been demonstrated to assist in a plethora of mechanisms to safeguard the cell from damage and allow it to survive, in cancer cells the same characteristics are devoted to the maintenance of the tumoral cell life and spreading, making HSP70 fit all the hallmarks of cancer [50]. In fact in cancer cells, HSP70 takes a place in many anti-apoptotic pro-survival pathways [51], i.e., HSP70 binds to DR4/5 (death receptor 4 and 5) [52], binds Bax [53], blocks JNK activity [54], stops the recruitment of pro-caspase 9 to the apoptosome [55], prevents the release to the cathepsins [56,57], etc. HSP70 is frequently aberrantly expressed in many solid tumors, as well as hematological malignancies with prognostic and therapeutic implications. HSP70 is largely expressed in hematological malignancies, including lymphoid diseases and chronic or acute myeloid leukemias, often associated with bad prognosis [58].

### 3.1. Acute and Chronic Leukemias

Acute lymphoblastic leukemia (ALL) is a heterogeneous disease bearing different clinical as well as biological features. The disease is characterized by the proliferation and the accumulation of lymphoid-lineage immature cells in the bone marrow, peripheral blood, lymphoid tissues and other compartments [59]. Acute myeloid leukemia (AML) is a clonal proliferation of hematopoietic stem cells, characterized by blocked or severely impaired differentiation and progressive accumulation of blasts in various stages of maturation [60]. Chronic myeloid leukemia (CML) is a chronic myeloproliferative disorder affecting the hematopoietic stem cell characterized by a leukocytosis of variable degree [61].

In 1992, the expression level of HSP70 was examined in cells from AML patients and demonstrated that the HSP70 protein was expressed in these cells, also in the absence of heat shock [62]. Later, the expression of HSPs was analyzed in permeabilized leukemic cells from both AML and CML patients by flow cytometry and showed that HSPs’ expression was significantly increased in AML versus CML [63]. Afterwards, the susceptibility to apoptosis of AML cells in vitro was correlated with intracellular expression of HSP70 and it was paradoxically observed that AML cells which expressed high levels of HSP70 were more prone to apoptosis [64]. Since the 2000s, a role has also been assigned for the form of HSP70 localized on the cell surface as a recognition signal for NK (natural killer) cytotoxic cells. It has been demonstrated, in fact, that HSP70^pos^ AML cells are killed by NK cells stimulated with low doses of IL-2 plus recombinant HSP70. Moreover, the investigation of the stimulation capacity of the TKD peptide (see 1.2) using PBMCs derived from AML patients as effector, and bone marrow-derived leukemic blast (HSP70^pos^) as target cells, indicated that membrane-bound HSP70 provides a target structure for TKD-activated PBMCs. Another piece of evidence resides in the fact that HSP70^pos^ K562 cells represent an activating ligand for IL-2/TKD-activated NK effector cells [65,66,67]. HSP70 membrane expression has also been associated with worse AML prognosis [68]. The plasma-circulating HSP70 was demonstrated to have a role in anti-tumor immune responses and its levels may reflect the severity of the disease. In AML and ALL patients, the HSP70 levels found in the plasma were significantly higher as compared to those in healthy controls, also being significantly correlated with lactate dehydrogenase (LDH) expression and white blood cells’ (WBC) counts, thus reflecting the overall tumor load [69]. Anti-HSP70 antibody and its antigen concentrations in the peripheral blood of AML patients were also evaluated to assess the utility of this determination, thus finding significantly higher anti-HSP70 antibody concentration compared to the control group. Patients who presented high levels of antigen but low levels of anti-HSP70 antibody had significantly shorter overall survival (OS) thus suggesting that the use of anti-HSP70 antibodies and HSP70 antigen could be valuable indicators of adverse prognosis in AML [70]. Still, in AML, high expression of *HSPA8* (that codes for the constitutive form of HSP70) has been observed to be associated with adverse clinical outcomes, this gene being identified as a potential marker for shorter OS in AMLs bearing a normal karyotype [71]. Several works have also been dedicated to the forms of HSP70 residing in particular cell compartments such as GRP78 of the endoplasmic reticulum or GRP75 in the mitochondrion. As a key component of the pro-survival axis of the unfolded protein response (UPR), GRP78 is highly expressed in relapsed B-lineage ALL, contributing to chemotherapy resistance of leukemic B-cell precursors [72]. In this context, literature data demonstrated an unrecognized vulnerability of pre-B cell-derived ALL cells to genetic or pharmacological inhibition of the UPR pathway thus establishing a mechanistic rationale for the treatment of children with pre-B ALL with agents that block the UPR pathway and induce ER stress [73]. In an effort to better characterize high- and standard-risk pediatric B-ALL patients at diagnosis, a study explored the role of surface (s) GRP78, thus reporting a distinctive cluster containing high levels of sGRP78, CD10, CD19, and CXCR4 in bone marrow samples obtained from high-risk patients. Moreover, circulating lymphoblastic leukemia cells were shown to express sGRP78 and CXCR4 [74]. Although upregulation of HSP70 has been demonstrated to be involved in tumor development in ALL, the molecular mechanism of HSP70 in ALL remains unclear. Guo et al. recently demonstrated that HSP70 is induced in leukocytes and monocytes from the blood of ALL patients and its suppression enhances apoptosis and inhibits cell proliferation by suppressing TAK1 and inducing Egr-1 [75]. In B- and T-leukemia cell lines, HSP70 is induced both at the cell surface and in the cytoplasm by anti-CD99 antibody, where this receptor is involved in various intracellular and extracellular processes such as adhesion, migration and apoptosis [76,77,78]. CD99 ligation enhances HSP70 transcription in ALL cells [79]. Different authors over the years have assigned to HSP70 a role as a potential therapeutic target and in reversing drug-resistance. Different strategies for targeting HSP70 have been proposed as monotherapy or in combination, especially with HSP90 inhibitors (i.e., 17-AAG or 17-DMAG). With regard to the latter statement, data from literature indicate that HSP70 induction attenuates the apoptotic effects of 17-AAG and, on the other hand, abrogation of HSP70 significantly enhances the anti-leukemia activity of 17-AAG [80,81]. Kaiser et al. investigated the in vitro anti-leukemic effects of pifithrin-μ, an inducible HSP70 inhibitor, in AML and ALL cell lines and in primary AML blasts, demonstrating that pifithrin-μ was effective in inhibiting cell viability at micromolar concentrations [82]. Moreover, in AML using VER-155008, a dose-dependent inhibition of cytokine-dependent AML cell proliferation and a pro-apoptotic effect have been demonstrated, this effect being enhanced in combination with HSP90 inhibitors [83]. Another study provided evidence that the xanthone beta-mangostin is effective in inducing apoptosis in the promyelocytic leukemia HL60 cell line, upregulating p53 and Bax and suppressing Bcl-2 and HSP70 genes in addition to arresting the cell cycle in vitro [84]. Another natural compound, parthenolide, targets HSP70, thus inducing heat shock response in THP-1 leukemia cells [85]. In 2021, Hu et al. discovered a novel HSP70 inhibitor with potent anti-tumor efficacy, named QL47, showing that HSP70 targeting might be a promising therapeutic method for the treatment of FLT3-ITD-positive AML, potentially able to overcome drug-resistance [86]. Since proteasome has been validated as a target of cancer therapeutics, the deubiquitinase (DUB) inhibitor VLX1570 was characterized in ALL at both UPR and protein translation levels. VLX1570 induced accumulation of polyubiquitinated proteins and increased expression of the chaperone GRP78 in ALL cells and also induced cleavage of PARP, meaning it induced apoptosis [87]. The same compound was demonstrated to have a potential anti-leukemic effect through the generation of ROS and induction of ER stress in both myeloid and lymphoid leukemia cell lines [88]. Thanks to CAR-T technology, a CAR-T directed against GRP78 found on AML blasts’ surface has been developed. GRP78-CAR T cells sequentially kill tumor cells and secrete cytokines with a potent anti-AML activity in vivo, this effect being improved by the Src kinases inhibitor Dasatinib [89]. In another study, the immunogenicity of AML and ALL cells was enhanced by transfection with the HSP70 gene of BCG (Bacille Calmette–Guérin). Short-term culture of those leukemia cells exhibited an increased number, no change in antigen expression, and enhanced immunogenicity with beneficial anti-leukemia effects [90]. Strategies indirectly targeting HSP70 have also been explored. CX-4945, the highly specific orally available ATP-competitive inhibitor of CK2α, induces apoptosis in T-ALL cell lines and T-lymphoblasts from patients. CX-4945 affects the UPR, as demonstrated by a significant decrease in the levels of GRP78 thus leading cells to apoptosis [91].

### 3.2. Chronic Lymphocytic Leukemia

Chronic lymphocytic leukemia (CLL) is a lymphoproliferative disorder characterized by the accumulation of small mature B lymphocytes due to both increased proliferation and defects in apoptotic mechanisms [92]. In 2010, a study investigated the localization of different HSPs (including HSP70) in B cells from patients with CLL and age-matched healthy subjects. Patients were found to differently express very high or very low levels of both sHSP70 and intracellular (i) HSP70 in CD5^+^/CD19^+^ neoplastic cells, although surface and intracellular datasets did not correlate. Levels of circulating HSP70 were found to be correlated with intracellular levels of HSP70 and were also found to be significantly lower in patients undergoing corticosteroid-containing regimens [93]. Moreover, GRP78 has been found to be expressed on the surface of CLL cells and associated with sMIC-A, a molecule which impairs NKG2D-mediated cytotoxicity [94]. Another study demonstrated that both lymphocytes and monocytes from CLL, and also from chronic myelomonocytic leukemia (CMML), showed high levels of total HSP70 expression versus healthy subjects, the majority of HSP70 in these tumors was determined to be expressed at the cell surface [95]. In CLL, it has been proposed that a combination of sub-lethal doses of chemotherapeutic agents and membrane fluidizing treatments, enhances drug efficacy and apoptosis in vitro. The treatment also resulted in a significant contemporary decrease in iHSP70 and increase in sHSP70, this localization affecting the cytotoxicity of doxorubicin [96]. The “molecular machine” HSP70, which inhibits cell death by stopping the recruitment of procaspase-9 to the Apaf-1 apoptosome, was found overexpressed in CLL cells following an RPPA (Reverse Phase Protein Array) study by us. In that manuscript, our group demonstrated a correlation between HSP70 levels and the response to chemo-immunotherapy (i.e., fludarabine and cyclophosphamide + rituximab or bendamustine + rituximab) in CLL. We inhibited HSP70 in CLL cells with pifithrin-μ (mentioned also in Section 3.1) a selective inhibitor of inducible HSP70, described to be able to affect more cancer than healthy cells and showing significant efficacy in vitro [97]. Later, we found that both HSP70 and its major regulator Heat Shock Factor 1 (HSF1) were overexpressed in CLL patients, correlated to poor prognosis and abnormally localized in the nucleus of leukemic B cells. The two proteins were strictly correlated to each other and their levels decreased consensually in those patients responding to in vivo therapeutic regimens (i.e., rituximab + bendamustine or the Btk inhibitor ibrutinib). Moreover, we found that the HSP70/HSF1 axis is a druggable target in CLL cells considering that neoplastic B cells underwent apoptosis when treated with HSP70/HSF1 axis inhibitors (i.e., fisetin, VER-155008, zafirlukast and KRIBB-11) [98]. In the same line of research, we addressed the role of the HSP70/HSF1 axis in the development of drug-resistance mediated by ibrutinib, finding an increase in both HSP70 and HSF1 proteins when the treatment was failing, and the disease was progressing. This finding suggests the involvement of HSP70 in mechanisms of drug-resistance. Moreover, we demonstrated that the use of HSP70/HSF1 axis inhibitors (i.e., resveratrol, honokiol, pterostilbene, and triacetyl-resveratrol), at different levels, could represent a novel rational therapeutic approach to overcome ibrutinib resistance in those patients who relapsed after this treatment [99]. Beside HSP70 itself, the role of its co-chaperone BAG3, has been characterized in CLL. Chen and colleagues showed that BAG3 mRNA levels were significantly higher in CLL than in healthy controls, with BAG3 levels in the drug-resistant group higher, as compared with the drug-responsive group [100]. The role of the BAG3 protein in leukemia cell survival and response to therapy has been reviewed in [101]. The mechanism of BAG3 in CLL has been addressed in Zhu et al. [102], this work highlighting that patients with higher BAG3 levels have a worse OS in ZAP-70 positive and p53 negative subgroups. Moreover, BAG3 has been demonstrated to inhibit cell apoptosis in primary CLL cells and its knock-down inhibited CLL cell migration. Recently, a study has been aimed at evaluating the effect of metabolic factors involved in invasive CLL on apoptotic factors. The obtained results showed a strong association among the expression of BAG3, GRP78 and HIF-1α with patients’ stages, highly correlated with their expression rate (both gene and protein). The increased expression of GRP78 and HIF-1α resulted in a BAG3 increase, as well as in disease progression [103].

### 3.3. Multiple Myeloma

Multiple Myeloma (MM) is a neoplastic disease involving plasma cells which proliferate and expand in the hematopoietic marrow and represent the cause of the typical multiple osteolytic lesions. Monoclonal plasma cells produce immunoglobulins, identical to each other, which migrate homogeneously during protein electrophoresis thus forming the characteristic monoclonal peak [104]. The first evidence of HSP70 research in MM was reported in 1989 when GRP78 was identified as the immunoglobulin heavy-chain-binding protein and was found expressed at a high level in the myeloma B-cell line NS-1 [105]. For other works concerning HSP70 in MM we had to wait for the first years of the twenty-first century. In those years, HSP70 inhibition has been demonstrated to reverse cell adhesion mediated- and acquired-drug resistance in MM. In MM cell lines and primary plasma cells, a role for HSP70 in the development of chemo-resistance was demonstrated through its enhanced expression after MM cells adhesion to both bone marrow stromal cells and fibronectin. Inhibition of HSP70 reduced adhesion, causing apoptosis of both acquired and de novo drug-resistant MM cells [106]. In the same context, later, GRP78 knock down was demonstrated to trigger dramatic changes in MM PC3 cell line morphology, thus decreasing their adhesion to osteoblasts and this dependent, at least in part, on a reduced N-cadherin expression [107]. In another study, beyond its own features, soluble HSP70 has been evaluated as a potential good biomarker of HSP90 inhibition as an alternative to cytosolic HSP70 [108]. As well as for other hematological diseases, inhibition of HSP70 (i.e., with triptolide and KNK437) in MM has been assessed to counteract the side effects of HSP90 inhibition and to enhance the apoptosis induced by it [109]. HSP72/73 were found to be overexpressed in MM and their knockdown or treatment with VER-155008 induced apoptosis of myeloma cells associated with a decreased protein level of HSP90-chaperone clients known to affect several oncogenic signaling pathways. Moreover, HSP70 knockdown/inhibition worked synergistically with the HSP90 inhibitor NVP-AUY922 in affecting myeloma cells’ viability [110]. The HSP70 inhibitor VER-155008 was used in combination with Bortezomib, this combination being demonstrated to induce a synergistic and remarkable MM cell apoptosis in vitro [111]. More recently, HSF1, the master regulator of HSP70, has been introduced as a possible therapeutic target (see also Section 3.2). Heimberger and colleagues reported that HSF1 is frequently overexpressed in INA-6 and MM.1S myeloma cell lines. HSF1 knockdown or its downregulation by triptolide, induces apoptosis in MM cell lines and in primary MM cells [112]. Moreover, HSF1 inhibition by KNK-437 in combination with bortezomib has been demonstrated to play additive effects on apoptosis induction in cells belonging to MM patients with poor prognosis [113]. A member of non-ATP-site inhibitors of HSP70, MAL3-101, has been examined for its anti-myeloma effect, thus discovering its pro-apoptotic function in MM cell lines in vitro and in vivo in a xenograft plasmacytoma model, as well as on primary tumor cells and bone marrow endothelial cells from myeloma patients. In addition, this inhibitor combined with the proteasome inhibitor MG-132, significantly potentiated its anti-myeloma effect [114]. Another piece of evidence that the targeting of HSP70 represents a good therapeutic approach which may be effective in the treatment of MM, was highlighted in L. Zhang et al. [115]. They demonstrated that the silencing of HSP70 increased Ig retention, decreased the ubiquitination required for proteasome degradation, and triggered multiple cellular responses (i.e., CDK4, C-RAF, HSF1, GRP78, LAMP-2A, and caspase-3) thus contributing to MM cell death. Moreover, in this study, VER-155008 was used alone or in combination with the HSP90 inhibitor 17-AAG. Again, the effects of VER-155008 in combination with bortezomib have been studied by Eugênio and colleagues [116]. A further HSP70 inhibitor, PET-16, was tested and it was found to induce apoptosis in MM cell lines at low micromolar doses (different from normal cells) also causing proteotoxic stress [117]. Treatment of myeloma cells with bortezomib increased GRP78 levels and activated GRP78-dependent autophagy. In particular, cells resistant to bortezomib showed a significantly upregulated GRP78. Co-treatment with metformin, an anti-diabetic agent, was demonstrated to suppress GRP78 and to enhance the pro-apoptotic effect of bortezomib [118]. Another study concluded that MM cells resistant to bortezomib treatment display a GRP78^high^/p21^high^/CDK6^low^/P-Rb^low^ profile, these markers identifying quiescent cells able to fuel a subsequent recurrence [119]. GRP78 is an appealing candidate for immunotherapeutic intervention as it is overexpressed at all myeloma stages and increased in patients with disease progression, especially in those patients with drug-resistance and extramedullary disease. The use of dexamethasone, PAT-SM6 (monoclonal antibody against sGRP78), and lenalidomide showed synergistic anti-MM effects in proliferation assays [120]. The role of GRP78 as a potential novel biomarker and/or therapeutic target in MM has been recently reviewed in Ninkovic et al. [121]. The immunomodulatory action of HSP70 has been recently highlighted since HSP70^pos^ exosomes are primarily found in the bone marrow of MM patients, contributing to IFNγ production and thus suggesting they play a crucial immunomodulatory action in the tumor microenvironment [122]. Another group found that the hDKK1–hHSP70 fusion vaccine could significantly suppress tumor growth of murine MM. A significant decrease in proliferation and an increase in apoptosis were also observed in the tumor tissues injected with the hDKK1–hHSP70 vaccine, demonstrating that xenogeneic homologous vaccination had great immunogenicity [123]. More recently, a decrease in HSP70 expression has been found in MM cells treated with a combination of gemcitabine + busulfan + melphalan + panobinostat + venetoclax (anti-Bcl-2) [124]. Allosteric HSP70 inhibitors, named JG compounds, have also been explored as myeloma therapeutics. They impact myeloma proteostasis by destabilizing the 55S mitoribosome thus suggesting JGs have the most prominent anti-myeloma effect through mitochondrial-localized HSP70 (i.e., GRP75) rather than through the inhibition of cytosolic HSP70 [125]. A very recent work identifies HSP70 family members as the “managers” of the molecular network of the proteasome machinery, except for controlling Nrf1/2. These results indicate the combination of HSP70 and Nrf1/2 inhibitors as promising therapeutic targets in MM [126].

### 3.4. Lymphomas

Non-Hodgkin’s lymphomas (NHL) are clonal lymphoproliferative diseases originating from B lymphocytes (80–85% of cases), T lymphocytes (15–20%) or natural killer lymphocytes (NK, rare). The classification of NHLs is currently based on the criteria proposed by the WHO and they are identified primarily on the basis of the cell of origin (B lymphocyte, T or NK) and therefore on morphological, immunophenotypic, genetic, and molecular criteria, integrated with the characteristics of the clinical presentation [127,128,129]. The information on HSP70 in the various lymphomas is incomplete and linked more to its modulation/role in the different pathways than to HSP70 inhibition itself, perhaps sometimes due to the rarity of some lymphomas. On average, leukemia cell lines analyzed, as compared to normal cells, exhibited surface HSP70 while mRNA expression was variable in the different cell lines [130].

#### 3.4.1. Diffuse Large B-Cell Lymphoma

A proteomic study, with subsequent validation, identified GRP78 as differentially expressed in non-GC (germinal center, higher expression) as compared to GC-DLBCL (diffuse large B-cell lymphoma) [131] thus highlighting the importance of this protein in lymphoma as well as in leukemia (see previous paragraphs). GRP78, in fact, is related to worse OS of DLBCL patients as well as related to bortezomib-resistance in DLBCL cell lines [132]. In activated B cell-like (ABC)-DLBCL, a common subtype of aggressive lymphoma typically refractory to therapies, an N-terminal misfolding mutation renders Blimp-1 (B lymphocyte-induced maturation protein-1) unstable. It has been demonstrated that HSP70 selectively escorts mutant Blimp-1 proteins to Hrd1 that, in turn, sequesters mutant Blimp-1 for cytoplasmic degradation. HSP70 inhibition (by VER155008) restores the function of mutant Blimp-1 and suppresses the growth of ABC-DLBCL xenografts [133]. In A20 and BL3750 lymphoma tumors in mice, the increased efficacy of accelerated local tumor irradiation was correlated with higher levels of tumor cell necrosis versus apoptosis and expression of “immunogenic cell death” markers, including HSP70 [134]. Recently, GRP75 was found to be highly expressed and correlated with resistance to rituximab-based therapy and poor survival in patients with DLBCL. In a mouse model, genetic depletion of GRP75 increased the activity of Rituximab indicating GRP75 is a novel target for the treatment of DLBCL [135].

#### 3.4.2. Mantle Cell Lymphoma

In 2013, the rise of HSP70 protein expression was highlighted in mantle cell lymphoma (MCL) as the reflection of the degree of cellular proteotoxic stress caused by the association of bortezomib plus CK2 inhibitors (i.e., CX-4945) in MCL cell lines [136]. Sehikara and colleagues proposed the simultaneous inhibition of XPO1 (nuclear transporter exportin-1) and mTOR signaling as a promising tool targeting pro-survival metabolism in MCL, this strategy inhibiting c-Myc, HSF1 and its target HSP70 [137]. Moreover, it has been demonstrated that MCL cells are sensitive to p97 inhibitors in vitro whose combination with HDAC6 inhibitors induces synergistic apoptosis by inducing the ER stress marker GRP78 [138].

#### 3.4.3. Hodgkin’s Lymphoma

HSP70 was also studied in patients with Hodgkin’s Lymphoma (HL) by an avidin–biotin immunoperoxidase complex technique, where staining resulting in HSP70 expression was found in 85% of the HL examined and the frequency of HSP70 positive cases was significantly higher than that of HSP70 negative cases. The association between HSP70 expression and HL thus appeared to be more frequent in patients with LD (lymphocyte depleted) and NS (nodular sclerosing) subtypes, although examples of HSP70-positive tumors were found in all histological subtypes [139]. HSC70 and HSP72 expression in HL, infected or not by Epstein–Barr virus (EBV) was also analyzed by the immunoperoxidase method in paraffin sections and demonstrated no differences according to clinical stage, treatment response or the presence of EBV. The pathological subtypes with the higher expression in lymphocytes were mixed cellularity and nodular sclerosis [140]. Later, using tissue microarrays, it was suggested there was a role for HSP70, and other apoptotic markers, in modulating the apoptosis in classical HL, mainly through the HSP70–HSP40 system, and in the stabilization of p53 [141]. As far as the ER counterpart of HSP70, GRP78, is concerned, it has been demonstrated that its expression was increased by LMP1 (latent membrane protein-1) transfection in HL cell lines. This study, first, evidenced that the expression of survival signals of ER stress (e.g., GRP78) was common in all histological subtypes of HL and was found in both EBV-positive and EBV-negative cases at a similar level [142].

#### 3.4.4. Other Lymphomas

In the U937 cell line (pleural effusion of a patient with histiocytic lymphoma), it has been observed that extracellular HSPs (including HSP70) increased the resistance to apoptotic cell death induced by hydrogen peroxide and diminished oxidative stress-mediated injuries [143]. The same cells, treated with TA, a synthetic thyroid hormone analog, significantly underwent apoptosis through the production of ROS, dysfunction of mitochondria, and activation of caspase cascade as well as the ER stress-marker GRP78 [144]. The Nalm-6 human B-lymphoid leukemia cell line treated with etoposide was proven to have chemoresistance characteristics after co-culture with HSP70-containing exosomes derived from bone marrow stromal cells (BM-SCs). Exosomes protected Nalm-6 cells from etoposide-induced apoptosis [145]. A study evaluated the effect of BMTP-78 (bone metastasis targeting peptidomimetic 78), a prototype drug composed by a GRP78-binding peptide fused to the pro-apoptotic enantiomer D(KLAKLAK)2, which disrupts the mitochondrial membrane causing cell death, in a panel of human leukemia and lymphoma cell lines. BMTP-78 was found to induce dose-dependent cytotoxicity in all samples tested [146]. Cells from primary effusion lymphoma (PEL), a non-Hodgkin’s B-cell lymphoma, were treated with arctigenin which markedly inhibited the proliferation of PEL by decreasing cellular ATP levels, disrupting mitochondrial membrane potential and triggering caspase-9-mediated apoptosis. In addition, arctigenin downregulated GRP78 thus corroborating the importance of this molecule in cell survival [147]. In the same cellular type, cell viability has been analyzed after application of HSP70 inhibitors, including pifitrin-μ, which demonstrated a dose- and time-dependent cytotoxic effect and induction of membrane permeabilization of lysosomes, transfer of cathepsin D in the cytosol, bid cleavage, and mitochondrial depolarization with release and nuclear translocation of apoptosis-activating factor [148]. As far as Burkitt’s lymphoma (BL) is concerned, the promoter of the human gene encoding the stress-responsive protein GRP78 was first isolated from BL cells by PCR in 1992 [149]. The role of HSP70 was also highlighted in the BL Raji cell line. In those cells, in fact, the inhibition of the JAK2/STAT3 signaling pathway by AG-490 [150] was proven to inhibit proliferation, to induce cell cycle arrest, and to promote oxidative stress and apoptosis via the downregulation of HSP70 [151]. In Raji and Daudi cell lines, the compound resveratrol, a natural phytoalexin, has been shown to induce anti-proliferation and apoptosis with a concomitant increase in expression levels of the GRP78 molecule [152]. This compound, resveratrol, has been used in the same HSPs’ context in CLL by Frezzato et al. [99]. Recently, it has been hypothesized that high HSP70 may predispose HIV-infected individuals to AIDS-non-Hodgkin lymphoma [153]. The ability of HSP70 to drive protein degradation has been also demonstrated in anaplastic large cell lymphoma (ALCL). In CD30+ NPM–ALK+ ALCL cells, in fact, HSP72 has been shown to regulate ALCL stress response and drug sensitivity [154,155]. In another model, it has been demonstrated that BAG-3 expression correlated with increased HSP70 expression in a subset of systemic T-cell lymphoma cases, except for cutaneous T-cell lymphoma (CTCL) [156]. As for the latter, HSP70 overexpression has been associated with chemoresistance of the disease to histone deacetylase inhibitors (HDACis; i.e., vorinostat). For this reason, a stable HSP70-knockdown CTCL cell line was established, thus confirming the influence of HSP70 reduction on the anti-tumor effects of vorinostat. Subsequently, quercetin was used to reduce HSP70 expression and enhance HDAC activity [157]. When a proteomic investigation was conducted in 33 lymphoid cell lines to identify novel therapeutic targets for enhancing the effects of HDAC inhibitors, HSP72 showed higher expression in cell lines exhibiting resistance to valproic acid, whose cytotoxic effect was enhanced by the treatment with KNK-437, an inhibitor of HSP72 [158]. In 2013, the group of Croce evidenced that HSP70 interacts with and regulates Tcl1 and protects it from degradation in leukemia and lymphomas [159].

## 4. Discussion and Conclusions

To date, inhibitors of HSP70 have not reached clinical practice, although a lot of data from the literature have as an object, the characterization and study of this protein. This lack is probably due to off-target effects, as HSP70 is a ubiquitous protein with multiple functions whose inhibition could also affect healthy cells. Attempts of a few clinical trials involving in some way HSP70 are reported in Table 2.

**Figure 3:**
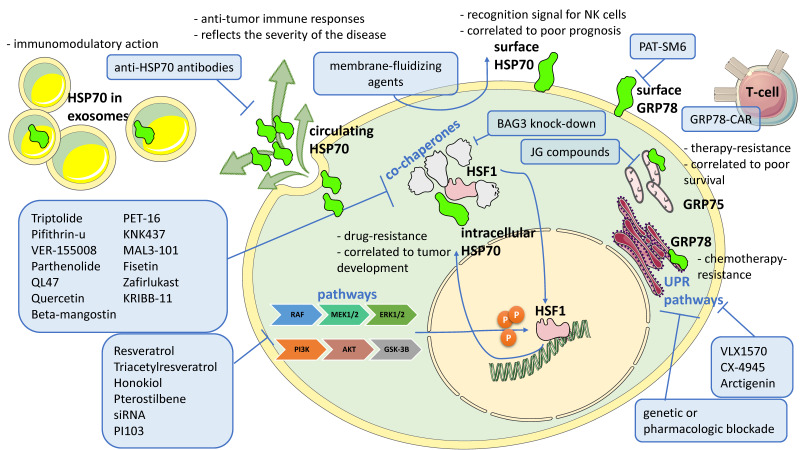
Overview on HSP70 localization, characteristics and proposed targeting. Different forms and localization of HSP70 (green fluorescent objects) are reported, for each of which some characteristics are also referred. Inhibitors or agents for HSP70 targeting named in this review, are listed in light blue boxes. Blue connectors are referred to inhibition (T-shaped lines) or promoting mechanism/activation (arrows). “P” inside orange circles is phosphorylation. CAR = chimeric antigen receptors; UPR = unfolded protein response.

In any case, the study of HSP70 and its inhibition is still very active in both solid tumors, such as brain [167], lung [168], colorectal [169], prostate [170] to name a few, and in other diseases such as cystic fibrosis [171]. Moreover, HSP70 has been taken into consideration in the immunotherapy context, as HSP70 derived from tumor cells does elicit tumor immunity by inducing specific anti-tumor immune response activating cytotoxic T cells. In addition, HSP70 is known to be a strong enhancer of antigen-presenting cells and therefore, upregulates pro-inflammatory cyto/chemokines’ production and strengthens the immunogenicity of antigens. HSP70 from *Mycobacterium tuberculosis* (mycHSP70) has been shown to exert a potent adjuvant effect in vaccination against both infectious agents and solid tumors. In the 38C13 murine lymphoma model, it has been observed that mycHSP70 plasmid prolongs survival of immunized mice challenged with a high number of tumor cells [172]. Another side of HSP70, that is the potential role of cytosolic and extracellular HSP70 as potential biomarkers for aGVHD (acute graft versus host disease) and its role in preclinical models, is discussed in [173]. Monitoring levels of HSP70 protein and/or anti-HSP70 antibody levels in the serum following HSCT might, in fact, be a useful diagnostic tool to predict the onset of GVHD. Moreover, it has been described that genetic or pharmacological modulation of the HSP70 expression may have therapeutic potential in the treatment of GVHD. Translation of HSP70 inhibition into the clinic is therefore highly desirable. The correct strategy will probably be represented by the use of low-dose HSP70 inhibitors in combination with other drugs. Moreover, it is of notice that HSP70 co-chaperones could themselves become therapeutic targets as they are often altered in cancer cells, too (see BAG3 in CLL). For the same reason, HSP70 transcription factor HSF1 could be a very adequate therapeutic target. Again, pathways that culminate in HSP70 modulation or activation are likewise druggable. Among others, in fact, an involvement of both PI3K/Akt and MEK1-2/ERK1-2 pathways has been described in the upregulation of the HSF1/HSP70 axis. PI3K/Akt inhibition by idelalisib not only induces apoptosis in leukemic B cells as expected, but it also reduces the expression levels of both HSP70 and HSF1 [98]. We ourselves used resveratrol, a natural polyphenol provided with anti-cancer properties through the modulation of different kinases (i.e., it inhibits Akt and activates Erk1/2), thus inducing the death of CLL cells by reducing HSP70 and HSF1 levels. Due to low bioavailability and poor potency of this molecule, other inhibitors with a structure and action like resveratrol, namely pterostilbene, triacetylresveratrol and honokiol, have been used and have been shown to be significantly effective in inducing apoptosis in CLL cells at concentrations lower than that used for resveratrol [99]. This approach that indirectly downmodulates HSP70, aims at reducing the unpredictable off-target effects that the direct inhibition of HSP70 protein have shown. In MM cells, inhibition of the PI3K/Akt/GSK3β pathway, with siRNA or PI103, decreased expression of HSF1 and downregulated both HSP70 and HSC70 expression. An addition to synergistic cytotoxicity was demonstrated when myeloma cells were treated with a combination of NVP-AUY922 and PI103 [110]. The study of HSP70 is not only a legacy of the past and research works on the promising potential of HSP70 as a therapeutic target have been produced recently. Results obtained in the different diseases should be observed as an overview and the knowledge obtained on one subtype should also be applied to another one (Figure 3). For all these reasons, and after further efforts of researchers, yes, HSP70—and surely its partners—could still be considered a therapeutic target for onco-hematologic diseases.

## Figures and Tables

**Table 1:** HSP70 family proteins.

CHROMOSOME	GENE	PROTEIN (and Alternative Names)	LOCALIZATION	INDUCIBLE
6p21.3	HSPA1A	**HSPA1A**, HSP70-1, HSP72, HSPA1, HSP70-1A, HSP70i	Cytosol, nucleus, cell. membrane, exosomes	Yes
6p21.3	HSPA1B	**HSPA1B**, HSP70-2, HSP70-1B	Cytosol, nucleus, exosomes	Yes
6p21.3	HSPA1L	**HSPA1L**, HSP70-1L, Hsp-hom, HSP70-1t, hum70t	Cytosol, nucleus	No
14q24.1	HSPA2	**HSPA2**, Heat shock 70kD protein-2, HSP70.2	Cytosol, nucleus, cell. membrane, exosomes	No
9q33.3	HSPA5	**HSPA5**, HSP70-5, BIP, GRP78, MIF2	ER, exosomes	No
1q23	HSPA6	**HSPA6**, HSP70-6, Heat shock 70KD protein 6 (HSP70B’)	Cytosol, exosomes	Yes
1q23.3	HSPA7	**HSPA7**, HSP70-7, Heat shock 70KD protein 7 (HSP70B)	Blood microparticles, exosomes	Yes
11q24.1	HSPA8	**HSPA8**, Hsp70-8, HSC70, HSC71, HSP71, HSP73	Cytosol, nucleus, cell. membrane, exosomes	No
5q31.1	HSPA9	**HSPA9**, HSP70-9, GRP75, HSPA9B, MOT, MOT2, PBP74, mot-2, mtHSP70, mortalin	Mitochondria, nucleus	No
10q26.12	HSPA12A	**HSPA12A**, HSP70-12A, FLJ13874, KIAA0417	Intracellular, exosomes	No
20p13	HSPA12B	**HSPA12B**, HSP70-12B, RP23-32L15.1, 2700081N06RIK	Endothelial cells, intracellular, plasma	No
21q11	HSPA13	**HSPA13**, HSP70-13, Stch	ER, exosomes, microsomes	No
10p13	HSPA14	**HSPA14**, HSP70-14, HSP70L1, MCG131990	Cytosol, cell. membrane	Yes

Information on HSP70 localization have been obtained from Kampinga HH et al. [12], Vega VL et al. [22], Gehrmann M et al. [25], Daugaard M et al. [32], Radons J [33], Tavaria M et al. [34], Mizzen LA et al. [35], Hu G et al. [36], Otterson GA et al. [37], Vostakolaei MA et al. [38], Espinoza MF et al. [39], Wan T et al. [40], and https://www.proteinatlas.org/ (accessed on 25 February 2023).

**Table 2:** Clinical trials about HSP70 in hematological malignancies. Data have been obtained from https://clinicaltrials.gov/ and PubMed (accessed on 25 February 2023).

CT.gov Identifier	Study Phase	Disease	Status	Aims	Location	Publication
NCT00030303 ^#^	I	CML	C	Determine the feasibility and toxicity of vaccination with autologous HSP70 in patients with chronic phase CML.	US	[160]
NCT00027144 ^#^	I	CML	C	Determine the response of the immune system of patients with CML to a vaccine made from their own tumor.	US	NA
NCT00058747 ^#^	II	CML	T	Investigate AG-858 (Autologous HSP70 Protein-Peptide Complex), in CML patients who are cytogenetically positive after treatment with Gleevec.	US/UK	NA
NCT00096005 ^#^	I	Lymphoma and Solid tumors	T	Determine the dose-limiting toxicity and maximum tolerated dose of 17-AAG and bortezomib in patients with advanced solid tumors or lymphomas and determine changes in biomarkers (e.g., HSP70)	US	NA
NCT00957736 ^#^	I	cGVHD in patients after donor stem cell transplant	T	Determine and define the biological basis of different subtypes of cGVHD using a targeted SNP (including HSP70) approach.	US	NA
NCT00175812 *	I/II	AML	C	Primary outcome measure: survival.	Norway	[161,162,163]
NCT00995332 *	I/II	AML	C	Determine survival as primary outcome measure.	Norway	[161,164]
NCT00098423 *	I	R/R Acute and Chronic Leukemias	C	Determine the maximum tolerated dose and toxicity of 17-AAG when administered with cytarabine and determine the effect of this regimen on client proteins in vivo and ex vivo in leukemic blasts.	US	[165]
NCT00457782 *	I	MM, CLL and NHLs	C	Determine the safety, tolerability and dose-limiting toxicities of KW-2478 and determine the maximum tolerated dose and recommended Phase 2 dose for patients.	UK	[166]

^#^ Clinical trials obtained directly from ClinicalTrials.Gov database with the following search criterium: “leukemia” as disease term and “HSP70” as other term. * Clinical trials obtained from ClinicalTrials.Gov database but the ID number was found in PubMed. C = completed; the study has ended normally, and participants are no longer being examined or treated (that is, the last participant’s last visit has occurred), as defined in CT.gov. T = terminated; the study has stopped early and will not start again (participants are no longer being examined or treated), as defined in CT.gov. CML = Chronic Myelogenous Leukemia; cGVHD = chronic Graft Versus Host Disease; SNP = Single Nucleotide Polymorphism; AML = Acute Myeloid Leukemia; R/R = Relapsed/Refractory; MM = Multiple Myeloma; CLL = Chronic Lymphocytic Leukemia; NHLs = non-Hodgking Lymphomas.

## Data Availability

Not applicable.
